# Auditory Emotion Word Primes Influence Emotional Face Categorization in Children and Adults, but Not Vice Versa

**DOI:** 10.3389/fpsyg.2018.00618

**Published:** 2018-05-01

**Authors:** Michael Vesker, Daniela Bahn, Christina Kauschke, Monika Tschense, Franziska Degé, Gudrun Schwarzer

**Affiliations:** ^1^Department of Developmental Psychology, Justus-Liebig-Universität Gießen, Giessen, Germany; ^2^Clinical Linguistics, Department of German Linguistics, Philipps-Universität Marburg, Marburg, Germany

**Keywords:** emotion processing, cross-modal integration, priming effects, emotion words, emotional facial expressions, developmental changes, categorization

## Abstract

In order to assess how the perception of audible speech and facial expressions influence one another for the perception of emotions, and how this influence might change over the course of development, we conducted two cross-modal priming experiments with three age groups of children (6-, 9-, and 12-years old), as well as college-aged adults. In Experiment 1, 74 children and 24 adult participants were tasked with categorizing photographs of emotional faces as positive or negative as quickly as possible after being primed with emotion words presented via audio in valence-congruent and valence-incongruent trials. In Experiment 2, 67 children and 24 adult participants carried out a similar categorization task, but with faces acting as visual primes, and emotion words acting as auditory targets. The results of Experiment 1 showed that participants made more errors when categorizing positive faces primed by negative words versus positive words, and that 6-year-old children are particularly sensitive to positive word primes, giving faster correct responses regardless of target valence. Meanwhile, the results of Experiment 2 did not show any congruency effects for priming by facial expressions. Thus, audible emotion words seem to exert an influence on the emotional categorization of faces, while faces do not seem to influence the categorization of emotion words in a significant way.

## Introduction

The perception of basic emotion has been studied extensively in several modalities, with faces and language being among the most extensively studied modalities (for a recent review see Schirmer and Adolphs, 2017). Increasingly, researchers are examining the interaction and mutual influence between these two modalities during the perception of emotions, especially when it comes to the interaction of auditory information and facial emotions (Filippi et al., 2017; Fugate et al., 2017). Examining the nature of this interaction is crucial if we want to understand emotion perception under naturalistic conditions, since both audible language and face processing are often used together during social interaction.

Another important aspect of the interaction between audible emotional speech and face processing is its course of development during childhood, which is an area that has not been extensively studied thus far beyond infancy. However, in the years beyond infancy as children increasingly learn the meaning of emotional words, such interactions can have a crucial impact on how they perceive emotions. It was thus the central aim of the present study to explore the development of the interaction between the modalities of audible speech and faces in the perception of basic emotion during childhood.

Past research on adults has already established that multi-modal audiovisual presentations of emotions, such as a recording of a visible person speaking emotion words, are recognized better than uni-modal presentations consisting of only the visual or only the audio signals (e.g., Paulmann and Pell, 2011). Further, in terms of looking at the interference effects between the audio and visual modalities of emotion perceptions, several groups of researchers have carried out experiments where participants had to correctly recognize a target based on only a single modality, while the other modality conveyed information which was incongruent with the target. For instance, studies by Carroll and Young (2005), Collignon et al. (2008), and Pell (2005) have shown that participants performed worse in the recognition of facial emotions when accompanied by or primed by incongruent emotional sound recordings (either simple sounds or pseudowords containing no semantic information). Additionally, some experiments have also shown similar effects of congruency between emotional faces and written emotion words (Carroll and Young, 2005; Fugate et al., 2017), which despite being limited to only the visual stimulus modality, further underscore the mutual influence of faces and language for the perception of emotion. A recent study (Filippi et al., 2017) also tested multi-modal perception of emotion using three channels of information, including two channels of auditory information, prosody and semantic meaning, and one channel of visual information, facial expressions. The authors found that incongruency between the attended channel and the two remaining disregarded channels produced a decrease in the accuracy of recognizing the target emotions. Thus, it seems fair to conclude that the cross-modal interference from incongruent information is an indicator of important mutual influences between the perception of emotion from faces and auditory speech.

Regarding the development of these abilities to integrate audible speech and facial expressions for the perception of emotion, the only developmental studies on this topic of which we are aware have focused on the period of infancy. Between the ages of 3 and 12 months infants begin to make associations between verbal and facial stimuli, showing recognition of matching between a speaker’s voice and facial identity, as well as between heard speech and the correct corresponding mouth movements, demonstrating an increasingly sophisticated integration of verbal and facial information on a general level (for a review, see Watson et al., 2014). With regards to the audio-visual perception of emotions in particular, studies have shown that by 7 months infants are already sensitive to congruency between speech and facial expressions as indicated by both a looking preference for congruent face-voice pairs (Walker, 1982; Walker-Andrews, 1986) and ERP correlates of detecting incongruence between the facial and audio stimuli (Grossmann et al., 2006), see (Grossmann, 2010) for a review.

However, little is yet known about how children develop in their ability to integrate these two types of information for emotion perception beyond infancy. Some studies (Robinson and Sloutsky, 2004; Jerger et al., 2009) have already shown that children from the age of 4 undergo significant changes in their weighting of audio and visual information, although these studies did not investigate emotion perception. For instance, using tasks with competing information from the visual and auditory modalities, Robinson and Sloutsky (2004) found that infants showed an overall auditory preference, 4-year-old children showed both auditory and visual preferences depending on the exact condition, and adults showed an overall visual preference. It therefore seems logical to hypothesize that the ability to integrate verbal speech and facial expressions for emotion perception may likewise exhibit changes during the period of middle childhood when children begin to attend school and significantly improve their ability to process basic facial expressions of emotion (De Sonneville et al., 2002; Gao and Maurer, 2010; Mancini et al., 2013; Rodger et al., 2015) and emotion words (Sylvester et al., 2016; Bahn et al., 2017b; Kauschke et al., 2017).

In the present study we therefore examined children from three age groups, 6, 9, and 12-year-olds, which covers the period of childhood between the starting of primary school and the beginning of early adolescence, when boys and girls undergo significant biological, cognitive, and social developments related to puberty (for a review see Meschke et al., 2012). Additionally, we also tested a group of college-aged adults.

In order to assess the developmental changes in the extent to which the facial and speech processing system influence one another on a very basic level of emotion perception, we used a cross-modal priming task which required participants from all age groups to simply categorize emotional faces and words as positive or negative. This task allowed us to simultaneously test the ability of emotional faces and words to interfere with one another across the visual and auditory modalities, how this effect might change with the increasing age of the participants, and whether these effects differ with respect to the emotional valence of the stimuli. We used a total of 48 positive and negative emotion words, and an equal amount of positive and negative emotional faces to alternatingly act as primes and targets in two cross-modal priming experiments. In Experiment 1, the categorization of positive and negative emotional face targets was primed by emotion words of either the same- (congruent condition) or opposite-valence (incongruent condition). Experiment 2 used the same basic structure and stimuli as Experiment 1, but with emotional faces priming the categorization of emotion word targets as positive or negative.

## Experiment 1

In Experiment 1 we investigated how children of 6, 9, and 12 years of age compared to adults in performing valence-based categorization of positive and negative emotional expression when they were primed with congruent or incongruent positive or negative emotion words.

### Materials and Methods

#### Participants

The group of 6-year-old children consisted of 12 boys and 14 girls, for a total of 26 participants with a mean age of 6.83 years, *SD* = 0.46 years. The group of 9-year-old children consisted of 12 boys and 13 girls, for a total of 25 participants with a mean age of 9.32 years, *SD* = 0.22 years. The group of 12-year-old children consisted of 12 boys and 11 girls, for a total of 23 participants with a mean age of 12.23 years, *SD* = 0.25 years. The group of adult participants consisted of 12 men and 12 women for a total of 24 participants with a mean age of 25.74 years, *SD* = 4.59.

#### Stimuli

The visual stimuli which acted as the targets for categorization consisted of 48 color photographs of faces provided by the Pell Laboratory (Pell, 2005). The photographs were of four male, and four female adults, all of whom had some experience in theater, and each of whom appeared in a total of six photos. Of these 48 photographs, 24 were of positive facial expressions (seven happy-surprised expressions with a widely opened mouth, five happy expressions with a slightly opened moth, and 12 happy expressions with a closed mouth), with each of the eight models appearing in three of these positive photos. The remaining 24 photographs were of negative facial expressions (6 angry, 6 fearful, and 12 sad), with each of the eight models appearing in three negative photos. Both the positive and negative categories of photos each consisted of 12 female and 12 male photographs.

The photographs were chosen in such a way that the positive and negative categories featured a variety of expressions, but still had very similar average ratings of arousal and valence as measured form a neutral point (i.e., the negative faces were as negative as the positive faces were positive) based on ratings made by adult participants in a separate study (Vesker et al., 2017) using a 5-point SAM scale (Bradley and Lang, 1994) for arousal, and a 7-point SAM scale for valence.

The auditory stimuli which acted as primes consisted of recordings of 24 positive and 24 negative German emotion words selected from the BAWL-R database (Võ et al., 2009). Each emotion category included five adjectives, nine verbs, and 10 nouns. The words were selected using values provided by the BAWL-R database such that the positive and negative emotion categories had similar average values of arousal and valence from a neutral point (from ratings by adults), as well as other linguistic variables such as the numbers of phonemes and morphemes (Bahn et al., 2017a). Each of the 48 words was recorded using a neutral prosody in two versions, one spoken by a female speaker, and the other by a male speaker. The average duration of both positive and negative word recordings was approximately 750 milliseconds.

#### Apparatus

All children performed two pre-tests to verify normal language and intelligence development. The Wortschatz- und Wortfindungstest WWT 6-10 (Glück, 2011) test for German vocabulary was used to assess language, and Raven’s Colored Progressive Matrices (CPM), a non-verbal pattern-matching test, was used to assess intelligence. Only children who achieved the age-appropriate scores on the pre-tests were included in the sample described earlier. Participants in the 6- and 9-year-old groups performed the pre-tests in a separate testing session prior to data collection for the experiment, while the 12-year-old children performed the pre-test prior to the experiment in a single session. Adult participants performed only the experiment itself in a single session.

Experimental data was collected using a laptop with a 15.6 inch screen using OpenSesame 2.9 (Mathôt et al., 2012), and all audio stimuli were played over headphones worn by participants. Responses were collected via button presses through a cardboard cutout which exposed only the relevant keys on the keyboard. The cardboard cutout also displayed stylized picture labels of a sun and a raincloud next to the response keys corresponding to the positive and negative responses, respectively.

#### Procedure

The experiment was conducted in accordance with the research ethics guidelines set out by the German Psychological Society (DGPs). The experimental procedures and informed consent protocols were approved by the Office of Research Ethics at the University of Giessen. Written informed consent was obtained from all adult participants, and the parents of the children prior to their participation in experiments.

Participants began with a training phase consisting of 12 randomly ordered trials after being instructed on the experimental procedure. Participants were instructed to listen to the prime word, and respond as quickly and accurately as possible to categorize the target face as being positive or negative. If the participant responded correctly to at least 11 out of the 12 practice trials, the practice phase would end immediately. If the participant responded to fewer than 11 of the 12 practice trials correctly, the 12 practice trials would be repeated once again, at which point the practice phase would end regardless of their score.

After the practice phase, the participants once again saw the instructions relating to the response key, and proceeded to perform 96 randomly ordered experimental trials (48 congruent and 48 incongruent) with a single short break at the midway point. The 96 experimental trials used the 48 words and faces described in the stimuli section above, while the 12 practice trials used a separate set of stimuli which did not appear in the experimental trials. Each of the 48 target faces appeared twice in the experimental trials, once primed by an emotion word with a congruent valence, and another primed by an emotion word with an incongruent valence. Assignment of the prime-word face-target pairs within the congruent and incongruent trial groups was randomized for each subject, and the speaker of the word prime was always matched to the gender of the target face.

After completing the experiment, participants in the children’s group got to choose a small present as a reward for their participation, while the adult participants received course-credits for their participation.

**FIGURE 1:**
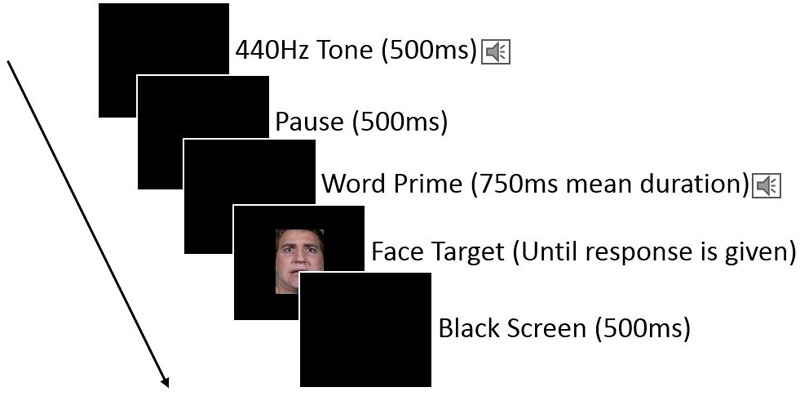
Illustration of an individual trial for Experiment 1, with emotion words priming the categorization of target faces.

Each trial began with a black screen and 500 ms long sine tone (440 Hz) was played over headphones to signal the trial start to the participants, followed by the another 500 ms pause, followed by the prime word being played over the headphones, followed by the appearance of the target face on the screen immediately after the end of the priming word. The target face remained on the screen until participants responded by pressing either the left or right response key (assignment of the keys to indicate “positive” or “negative” was randomly assigned for each subject at the start of their testing session). After participants pressed one of the response keys, the target face disappeared leaving a black screen for 500 ms until the start of the next trial. See **Figure 1** for an illustration.

#### Data Analysis

Data was analyzed on a per-trial basis with regards to accuracy and response time, with trials from participants who failed reached a minimum overall accuracy of 60% on experimental trials being excluded. Data from trials with a response time longer than an upper boundary calculated for each participant (two standard deviations over the mean response time) were also excluded to avoid outliers which may have resulted from participants being distracted. Accuracy was analyzed for all trials which passed the above exclusion criteria, while response time was analyzed for only the trials which passed the exclusion criteria and where the correct response was given.

### Results

#### Accuracy

A 4 × 2 × 2 ANOVA was carried out on the per-trial accuracy data, with age (6, 9, 12 years, and adults), face-valence (positive and negative), and congruency (congruent and incongruent) as the independent variables.

**FIGURE 2:**
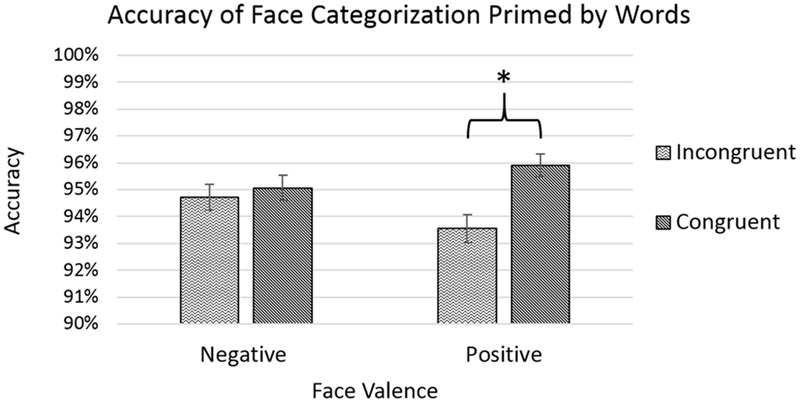
Accuracy rates for categorizing face targets after priming by emotion words. Error bars represent standard error, and a star indicates a significance level of *p* < 0.05.

The analysis showed significant main effects of age [*F*(3,8781) = 11.990, *p* < 0.001] with accuracy improving with increasing age, and of congruency [*F*(1,8781) = 8.491, *p* < 0.01], with congruent trials showing a greater average level of accuracy. The effect of congruency was qualified by a significant interaction between congruency and face-valence [*F*(1,8781) = 4.674, *p* < 0.05], with *post hoc* pairwise comparisons showing that congruent trials only differed significantly from incongruent trials for positive faces (*p* < 0.001), but not for negative faces (*p* = 0.596), see **Figure 2**. Please see **Appendix A** for a non-parametric analysis of the accuracy data for this experiment.

#### Response Time

A second 4 × 2 × 2 ANOVA was carried out on the per-trial response time data of correctly responded trials, with age (6, 9, 12 years, and adults), face-valence (positive and negative), and congruency (congruent and incongruent) as the independent variables.

**FIGURE 3:**
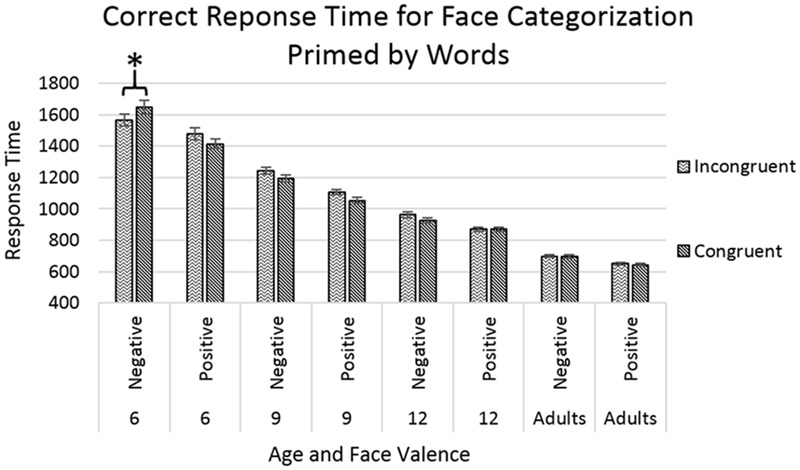
Response time for correct trials categorizing face targets after priming by emotion words. Error bars represent standard error, and a star indicates a significance level of *p* < 0.05.

The analyses showed main effects of age [*F*(3,8324) = 922.064, *p* < 0.001] with response time decreasing with increasing age, and of face-valence [*F*(1,8324) = 74.719, *p* < 0.001], with positive-face trials showing faster response times than negative-face trials. The effect of face-valence was qualified by a significant interaction between age and face-valence [*F*(3,8324) = 4.582, *p* < 0.01], with the difference between the response times for positive-face and negative-face trials being the greatest in the younger age groups, and diminishing with increasing age. An additional significant triple interaction was found between the factors of age, face-valence, and congruency [*F*(3,8324) = 2.710, *p* < 0.05], with all participants showing faster response times for congruent trials compared to incongruent trials except the 6-year-old group in the negative face trials, where children showed faster response times for incongruent trials (*p* < 0.05), see **Figure 3**. In other words, the 6-year-old children showed faster response time when primed by positive words, regardless of the valence of the target face.

### Discussion

With respect to accuracy, we found a general improvement in the accuracy of responses with increasing age. Additionally, our findings showed that all participants made more errors in categorizing positive faces when primed with incongruent negative words than with congruent positive words. Meanwhile, negative faces did not differ in categorization accuracy regardless of prime congruency. In other words, negative word primes proved to be particularly effective at interfering with the categorization of faces, as participants made more errors when negative word primes were incongruent with the target face.

The measures of response time for correct responses showed an overall increase in speed with increasing age, and that positive faces were generally categorized faster than negative faces, with this difference being most apparent in the younger age groups. Additionally, all age groups responded faster to congruent trials relative to incongruent trials except for the 6-year-olds, who showed faster response time when they were primed by positive words, regardless of congruency with the target face. It therefore appears that the 6-year-olds display a positivity bias in terms of their sensitivity to emotion word primes.

## Experiment 2

In Experiment 2 we examined how children at the same ages as in Experiment 1 and adults categorized positive and negative audible emotion words according to valence when they were primed with congruent or incongruent positive or negative emotional expressions.

### Materials and Methods

#### Participants

A new cohort of participants was recruited for Experiment 2, with no participants having participated in both experiments. The group of 6-year-old children consisted of eight boys and 14 girls, for a total of 22 participants with a mean age of 6.75 years, *SD* = 0.37 years. The group of 9-year-old children consisted of 11 boys and 12 girls, for a total of 23 participants with a mean age of 9.59 years, *SD* = 0.30 years. The group of 12-year-old children consisted of 11 boys and 11 girls, for a total of 22 participants with a mean age of 12.07 years, *SD* = 0.33 years. The group of adult participants consisted of 12 men and 12 women for a total of 24 participants with a mean age of 22.82 years, *SD* = 2.66.

#### Stimuli

The same auditory and visual stimuli were used as in Experiment 1, but with the visual stimuli now acting as primes, and the auditory stimuli acting as targets.

#### Apparatus

All the same tests, software, and equipment were used as in Experiment 1.

#### Procedure

The overall experimental procedure was identical to the one used in Experiment 1, except that now the faces served as primes, and the emotion words served as targets for categorization, and the instructions were changed to reflect this new testing procedure.

**FIGURE 4:**
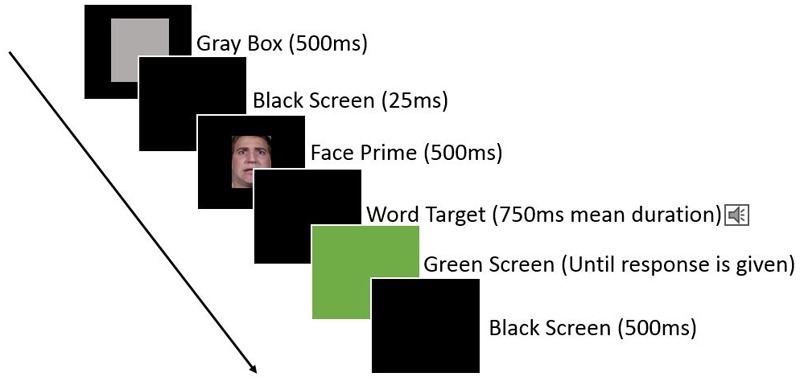
Illustration of an individual trial for Experiment 2, with faces priming the categorization of target emotion words.

Each trial began with the appearance of a gray box slightly larger than the prime face image in the center of the screen for 500 ms, followed by a black screen for 25 ms, followed by the appearance of the prime face for 500 ms in the center of the screen, followed by a black screen and the target word being played over headphones, followed by a green screen to signal to the participants that they may respond (this was to ensure that participants would listen to the entire word before responding). The screen remained green until participants responded by pressing either the left or right response key (assignment of the keys to indicate “positive” or “negative” was randomly assigned for each subject at the start of their testing session) through a cardboard cutout which exposed only the relevant keys on the keyboard. After participants pressed one of the response keys, the screen returned to black for 500 ms until the start of the next trial. See **Figure 4** for an illustration.

#### Data Analysis

The same data analysis procedure was followed as for Experiment 1.

### Results

#### Accuracy

A 4 × 2 × 2 ANOVA was carried out on the per-trial accuracy data, with age (6, 9, 12 years, and adults), word-valence (positive and negative), and congruency (congruent and incongruent) as the independent variables.

**FIGURE 5:**
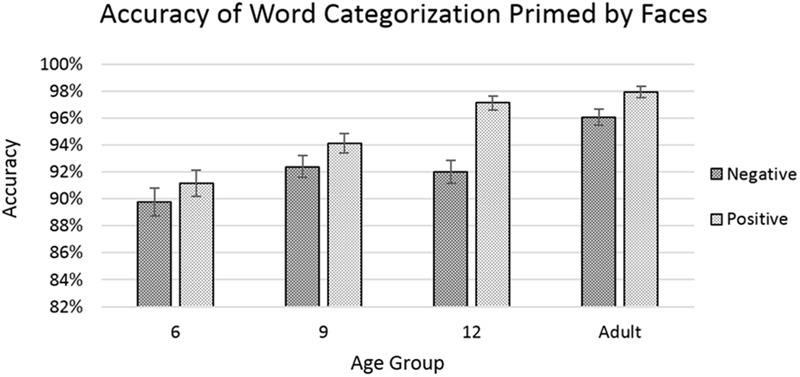
Accuracy rates for categorizing word targets after priming by faces. Error bars represent standard error. No significant effects or interactions were found involving the factor of congruency.

The analysis showed significant main effects of age [*F*(3,7949) = 25.360, *p* < 0.001] with accuracy improving with increasing age, and of word-valence [*F*(1,7949) = 22.685, *p* < 0.001], with positive-word trials showing a greater average level of accuracy than negative-word trials (**Figure 5**). The effect of world-valence was qualified by a significant interaction between age and word-valence [*F*(3,7949) = , *p* < 0.05], with the 12-year-old group showing the greatest difference between positive-word and negative-word trials. Please see **Appendix B** for a non-parametric analysis of the accuracy data for this experiment.

#### Response Time

A second 4 × 2 × 2 ANOVA was carried out on the per-trial response time data of correctly responded trials, with age (6, 9, 12 years, and adults), word-valence (positive and negative), and congruency (congruent and incongruent) as the independent variables.

**FIGURE 6:**
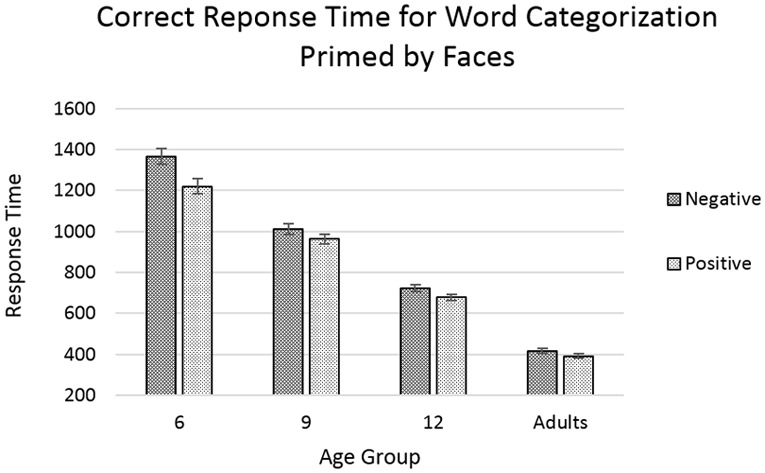
Response time for correct trials categorizing word targets after priming by faces. Error bars represent standard error. No significant effects or interactions were found involving the factor of congruency.

The analyses showed main effects of age [*F*(3,7468) = 528.870, *p* < 0.001] with response time decreasing with increasing age, and of word-valence [*F*(1,7468) = 16.568, *p* < 0.001], with positive-word trials showing faster response times than negative-word trials (**Figure 6**). The effect of face-valence was qualified by a marginally significant interaction between age and word-valence [*F*(3,7468) = 2.500, *p* = 0.058], with the difference between the response times for positive-word and negative-word trials being the greatest in the younger age groups, and diminishing with increasing age.

### Discussion

Our findings for the categorization of emotion words primed by faces seem to show general improvements in both speed and accuracy with increasing age. We also see that positive words are generally categorized faster and more accurately than negative words. Most notably, we did not find any main effects or interaction involving the factor of congruency, which indicates that the face primes did not significantly impact the categorization of emotion words.

## General Discussion

Our study was conducted with the goal of answering two primary questions. First, how do spoken words and facial expressions influence each other’s perception in a cross-modal primed categorization task? And second, how do these influences change as children increase in age through to adulthood, if at all? In Experiment 1 participants were tasked with categorizing photographs of emotional faces as positive or negative as quickly as possible after being primed with either an audibly presented positive or a negative emotion word in either a valence-congruent or incongruent fashion. In Experiment 2, we did the opposite and used faces as primes and words as the targets to be categorized as positive or negative in emotional valence.

With regards to the first question, we found that face primes did not influence the perception of words, as there were no congruency effects in Experiment 2. By contrast, words did influence the perception of faces, especially positive faces, as all participants showed a higher level of accuracy when positive faces were primed with positive words versus negative words in Experiment 1. Thus, it seems that audibly presented positive words have a greater influence on the perception of faces than vice versa. This finding bears a resemble to the “auditory dominance” commonly observed when subjects are required to perform tasks such as those requiring the perception of temporal sequences using competing information from visual and auditory modalities (Repp and Penel, 2002; Sloutsky and Napolitano, 2003; Kato and Konishi, 2006; Burr et al., 2009; Robinson and Sloutsky, 2010; Sandhu and Dyson, 2012). One possible reason for the apparent auditory dominance in the cross-modal perception of emotion stimuli in our study could be that the priming approach we used relies on automatic vigilance and processing of affective stimuli. Researchers have long theorized that this automatic processing of affective information may have arisen as a mechanism to enable the rapid avoidance of threats (Pratto and John, 1991; Hermans et al., 1994; Wentura et al., 2000). In this context it makes sense for hearing to dominate sight, since hearing allows one to be vigilant against potential threats in all directions, whereas sight only allows for vigilance toward what is in the observer’s visual field. Our findings seem to fit this hypothesis since only the negative words primes led to an increase of errors in incongruent trials.

Regarding the second question on developmental changes, our data for the speed of correct responses in Experiment 1 suggested that unlike all the older participants in our study, who would respond faster when the prime and target were congruent, the 6-year-olds would always respond faster when primed by positive words, regardless of congruence. This suggests that young children are particularly sensitive to positive words, which seems to drive them to make a faster response, either by allowing for the faster processing of affect-related information, or by increasing the children’s confidence. This agrees with the overall finding of a “positivity” bias in younger children in our data, as both experiments showed that although the ability to categorize both positive and negative faces and words improves with age, younger children are initially faster in categorizing positive words and faces compared to negative words and faces. This advantage for categorizing positive emotional stimuli faster than negative emotional stimuli decreases with age, and by adulthood, the observable difference between categorizing positive and negative stimuli is greatly reduced. This finding does not come as a surprise, as it has been observed in the literature that positive faces seem to be processed better when the task at hand requires participants to identify or categorize the stimuli as in our case, unlike tasks which require participants to detect them amongst distractors, which tend to favor negative stimuli (for a review see Nummenmaa and Calvo, 2015).

## Conclusion

Our study has shown that when it comes to the interaction of auditory speech and face perception in the processing of emotional stimuli, speech seems to have a greater impact on face perception than vice versa. Additionally, we have shown that of the age groups in the present study, 6-year-old children are particularly sensitive to positive word primes, to the extent that they make correct responses to subsequent target faces faster than when they are preceded by negative word primes, even when those target faces are themselves negative.

### Limitations

Our study was limited by the adult participants being restricted to younger college-aged adults. In light of research showing that the positivity bias which we observed to decrease from childhood to adulthood re-emerges in older adults, it would be interesting to see if the increased sensitivity to positive word primes which we observed in our youngest age group likewise reemerges in old age. Such an experiment could also determine how age-related declines in hearing and visual sensitivities influence the weighing of conflicting emotional information from faces and words. Additionally, our stimuli were limited to photographs of adults, and all the words were likewise spoken by adults. It would thus be informative for future studies to investigate the effects reported here with photographs and recordings of children, adolescents, or older adults, as their social relevance to the participants may be rather different depending on their perceived status as being below, above, or at the peer level of participants. Finally, our study was somewhat limited by the scope of tested sample. In order to confirm the generalizability of our findings to the larger population, a larger study should be conducted with additional age bands and more participants within each band.

## Notes

The raw accuracy and response time data for Experiments 1 and 2 will be made available to all readers on the Zenodo platform.

## Author Contributions

MV was involved in the experimental design, programming of the experiments, data analysis, and the writing of the manuscript. DB was involved in the experimental design, organization of data collection, and supervision of research assistants during data collection. CK was involved in the development of the original research topic and the experimental design. MT was involved in organizing data collection and supervision of research assistants during data collection. FD was involved in the development of the original research topic and the experimental design. GS was involved in the development of the original research topic, the experimental design, data analysis, and editing of the manuscript.

## Conflict of Interest Statement

The authors declare that the research was conducted in the absence of any commercial or financial relationships that could be construed as a potential conflict of interest.

## Appendix A

A non-parametric ANOVA-type statistic was calculated for the accuracy results of Experiment 1 to complement the standard ANOVA already described in the paper. This analysis was carried out in R using the rankFD package accessed from the CRAN repository. The analysis used the same factors as the ANOVA reported earlier for the accuracy measures of Experiment 1: A 4 × 2 × 2 design using the per-trial accuracy data as the dependent variable, with age (6, 9, 12 years, and adults), face-valence (positive and negative), and congruency (congruent and incongruent) as the independent variables.

Results were very similar to those of the ANOVA described earlier for the accuracy measures of Experiment 1 with significant main effects of age [*F*^a^(3,8024) = 11.558, *p* < 0.001], congruency [*F*^a^(1,8024) = 8.611, *p* < 0.01], and a significant interaction between age and congruency [*F*^a^(1,8024) = 8.611, *p* < 0.01].

## Appendix B

A non-parametric ANOVA-type statistic was calculated for the accuracy results of Experiment 2 to complement the standard ANOVA already described in the paper. This analysis was carried out in R using the rankFD package accessed from the CRAN repository. The analysis used the same factors as the ANOVA reported earlier for the accuracy measures of Experiment 2: A 4 × 2 × 2 design using the per-trial accuracy data as the dependent variable, with age (6, 9, 12 years, and adults), word-valence (positive and negative), and congruency (congruent and incongruent) as the independent variables.

Results were very similar to those of the ANOVA described earlier for the accuracy measures of Experiment 2 with significant main effects of age [*F*^a^(3,6331) = 25.079, *p* < 0.001], word-valence [*F*^a^(1,6331) = 21.666, *p* < 0.001], and a marginally significant interaction between age and word-valence [*F*^a^(3,6331) = 2.582, *p* = 0.057].
